# Cory’s Shearwater (*Calonectris borealis*): Exploring Normal Head Anatomy through Cross-Sectional Anatomy, Computed Tomography and Magnetic Resonance Imaging

**DOI:** 10.3390/ani14131962

**Published:** 2024-07-02

**Authors:** Alejandro Morales-Espino, Soraya Déniz, Pablo Paz-Oliva, Natalia Roldán-Medina, Mario Encinoso, Francisco Suárez-Cabrera, Jose Raduan Jaber

**Affiliations:** 1 IVC Evidensia Los Tarahales, 35013 Las Palmas, Gran Canaria, Spain; 2 Hospital Clínico Veterinario, Facultad de Veterinaria, Universidad de Las Palmas de Gran Canaria, Trasmontaña, Arucas, 35413 Las Palmas, Gran Canaria, Spain; 3 Department of Morphology, Facultad de Veterinaria, Universidad de Las Palmas de Gran Canaria, Trasmontaña, Arucas, 35413 Las Palmas, Gran Canaria, Spainnatalia.roldan101@alu.ulpgc.es (N.R.-M.);

**Keywords:** computed tomography, magnetic resonance imaging, head, anatomical sections, seabirds, Cory’s shearwater

## Abstract

**Simple Summary:**

This investigation describes the intricate anatomy of the central nervous system (CNS) and its related structures in Cory’s shearwater, employing anatomical cross-sections and imaging methodologies like computed tomography (CT) and magnetic resonance imaging (MRI). This study represents the inaugural comprehensive portrayal of the head anatomy in Cory’s shearwater, and the findings derived from this study promise to enrich future research in both the anatomical and pathological fields of closely related species.

**Abstract:**

Cory’s shearwater, or *Calonectris borealis*, stands out as a symbolic figure in the world of seabirds, playing a crucial role in marine ecosystems globally. Belonging to the Procellariidae family, it is singularized by its imposing wingspan and intricate migration patterns connecting it to various regions from the North Atlantic to the Pacific. Its role in the marine food chain, specialized diet and adaptation for nesting in the Canary Archipelago underscore its ecological importance. However, Cory’s shearwater also faces important threats, such as the invasion of foreign species, highlighting the need for its conservation. Among the conservation issues, studies on its biology, the main threats it faces and its normal anatomy are essential to preserve marine biodiversity. Additionally, a variety of imaging techniques, such as computed tomography and magnetic resonance, facilitates the understanding of the bird’s neuroanatomy and opens future research possibilities in comparative neuroscience. Moreover, this approach proves particularly relevant given the increasing attention these seabirds receive in environments such as zoos, rehabilitation centers and their natural habitat, where veterinarians play a crucial role in their care and well-being.

## 1. Introduction

Cory’s shearwater (*Calonectris borealis*) is a remarkable seabird known for its long-distance migrations and significant ecological role within the Canary Islands’ ecosystem [1,2,3,4]. Belonging to the distinguished Procellariidae family, it stands out for intricate migration patterns linking it to various regions worldwide, from the North Atlantic to the Indian and Pacific Oceans, and it occupies marine and coastal habitats [1,2,3,4]. During their migratory journeys, Cory’s shearwater populations trace routes covering significant distances, feeding and reproducing in a delicate balance with their marine environment. This migratory pattern reveals the complexity of their connection to oceanic ecosystems, where species survival and reproduction are intrinsically linked to the availability of food reserves [1,4]. As a vital component of the marine food chain, the shearwater deploys a specialized diet, focusing primarily on small fish, crustaceans and cephalopods. Its presence, therefore, contributes to the dynamics and stability of oceanic ecosystems, exerting a significant impact on the regulation of prey populations [1]. However, the importance of Cory’s shearwater transcends its direct ecological function. These seabirds, through their behavioral patterns and distribution, emerge as valuable bioindicators of marine environmental health [3,4]. Changes in their migration, reproduction, or abundance can serve as early warning signals for potential environmental threats, such as food scarcity or contaminants in the ocean [1,5,6]. In this context, the protection of nesting habitats, sustainable fisheries management and the mitigation of anthropogenic threats emerge as essential elements in conservation efforts to ensure the ongoing well-being of this seabird species [4]. Among these conservation issues, studies on its biology, the main threats it faces and anatomical investigations are essential for preserving marine biodiversity, since they elucidate the underlying mechanisms of avian physiology, and for comprehending the evolutionary adaptations that have allowed birds to inhabit a diversity of ecological niches [7,8,9,10,11,12,13,14,15,16,17,18,19,20,21,22,23,24,25,26,27,28,29]. 

Despite its ecological importance, there is a notable gap in the research concerning the neuroanatomy and cranial characteristics of this species using modern imaging diagnostic techniques. However, the revolution brought about by modern imaging techniques has marked a milestone in exploring this doctrine. These imaging techniques have already been employed in research on the nervous system of birds [30,31,32,33,34,35,36,37,38], but to date, specific research on the CNS and associated structures of Cory’s shearwater has not been conducted [32,38,39,40]. Therefore, this study aimed to describe the CNS and its associated structures through anatomical cross-sections and computed tomography and magnetic resonance imaging techniques. 

## 2. Materials and Methods

### 2.1. Animals

In this research, we utilized a cohort of 10 juvenile Cory’s shearwater specimens, characterized by an average mass of 0.520 kg (with a range of 0.480–0.820 kg) and an average length of 42 cm (with a range of 45–56 cm) from beak to tail base. These avian subjects were sourced from the Consejeria de Área de Medio Ambiente, Clima, Energía y Conocimiento of the Cabildo Insular de Gran Canaria, following strandings attributed to artificial lights, which have been recognized as a relevant threat to biodiversity conservation [3]. While most specimens were received post mortem, those initially alive but succumbing due to their weakened state were promptly preserved via freezing for subsequent CT and MRI procedures. It is important to emphasize that no animals were intentionally sacrificed or captured solely for scientific research.

### 2.2. CT Technique

For the CT assessment, we allowed our avian subjects to thaw at room temperature for 12 h. Sequential transverse CT images were obtained using a 16-slice helical CT scanner (Toshiba Astelion, Canon Medical System^®^, Tokyo, Japan). The birds were positioned symmetrically in dorsal recumbency on the stretcher with a craniocaudal entry. We used a standard protocol with the following parameters: 120 kVp, 80 mA, 512 × 512 acquisition matrix, 1809 × 858 field of view, a pitch of 0.94 and a gantry rotation of 1.5. The acquired images had a slice thickness of 0.6 mm. To enhance the visualization of various anatomical structures using CT, we employed different CT window settings by adjusting the window widths (WWs) and window levels (WLs): a bone window setting (WW = 1500; WL = 300), a soft tissue window setting (WW = 248; WL = 123) and a lung window setting (WW = 1400; WL = −500). No significant differences in CT density or anatomy were observed in the heads of the avian specimens utilized in this study. 

### 2.3. MRI Technique

The ten Cory’s shearwater specimens underwent imaging at Los Tarahales Veterinary Hospital (Las Palmas, Canary Islands, Spain). Imaging data were acquired using a Canon Vantage Elan 1.5 T imaging system (Toshiba, Vantage Elan, Japan), utilizing the following sequences: T1-weighted (T1W) sequences in the transverse plane (TR: 634 ms, TE: 10, FOV: 1809 × 829, slice thickness: 2 mm, matrix size: 192 × 160), T2-weighted (T2W) sequences in the transverse plane (TR: 4769 ms, TE: 120, FOV: 1809 × 829, slice thickness: 2 mm, matrix size: 192 × 224), T2W sequences in the dorsal plane (TR: 5271 ms, TE: 120, FOV: 1809 × 829, slice thickness: 2.5 mm, matrix size: 240 × 192) and T2W sequences in the sagittal plane (TR: 4450 ms, TE: 120, FOV: 1809 × 829, slice thickness: 2.9 mm, matrix size: 224 × 224). Additionally, we obtained enhanced spin-echo sequences in the dorsal, transverse and sagittal planes. The resulting MRI images exhibited a slice thickness of 2–3 mm.

### 2.4. Macroscopic Anatomical Sections

After imaging, the birds were preserved by freezing at −80 °C for 72 h. Following this preservation process, we sliced five specimens into transverse and dorsal sections, each 1 cm thick, utilizing an electric band saw. These sections underwent careful irrigation with water to eliminate any artefacts, such as feathers, which were removed using Adson forceps and identified, before being photographed on both sides to facilitate the precise identification of anatomical cross-sections and CT and MRI images. This dissection step greatly aided in achieving accurate identification and correlation with the CT and MRI images.

### 2.5. Anatomic Evaluation

To enhance the accurate identification of relevant structures within Cory’s shearwater heads, we selected anatomical cross-sections that closely matched the CT and MR images. Extensive reference materials, including textbooks, bird anatomy literature and bone preparations from other seabird specimens, were consulted to aid this endeavor [30,39,40,41,42,43,44]. Furthermore, to ensure the meticulous interpretation of cranial structures, we relied on anatomical preparations provided by the Department of Anatomy at the Faculty of Veterinary Medicine, University of Las Palmas de Gran Canaria. These supplementary resources significantly enriched our understanding and accuracy in the anatomical interpretation.

## 3. Result

**Figure 1:**
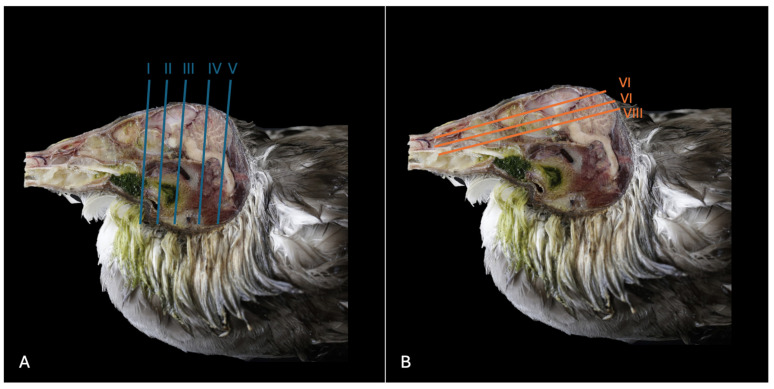
Sagittal cross-section images of the head of a Cory´s shearwater (*Calonectris Borealis*). The vertical (**A**) (labelled with blue) and horizontal (**B**) (labelled with orange) lines correspond to the approximate levels of the respective transverse and dorsal slices.

**Figure 2:**
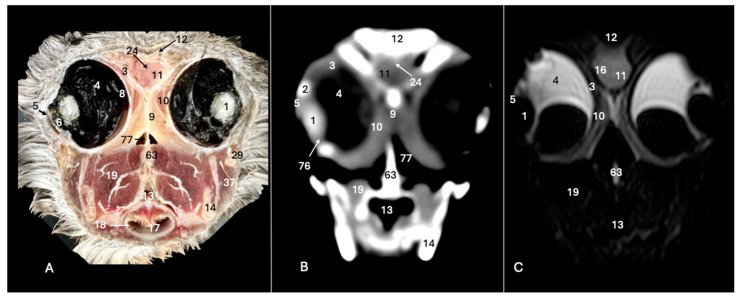
Anatomical cross-section (**A**), soft tissue CT window (**B**) and T2-weighted MR (**C**) transverse images of the Cory’s shearwater head at the level of the eyes, corresponding to line I in Figure 1. 1: *Lent cristali*; 2: sclerotic ossification; 3: sclera; 4: *camera vitrea bulbi*; 5: cornea; 6: *camera anterior bulbi*; 9: *septum interorbitalis*; 10: extraocular muscles; 11: olfactory bulb; 12: *os frontale*; 13: pharynx; 14: mandible; 16: cerebral hemisphere; 17: trachea; 18: tracheal ring; 19: *musculus pterygoideus pars ventralis*; 24: *fissura longitudinalis cerebri*; 29: *processus postorbitalis*; 37: *musculus adductor mandibulae externus*; 63: parasphenoid rostrum; 76: ciliary body; 77: *choana*.

**Figure 3:**
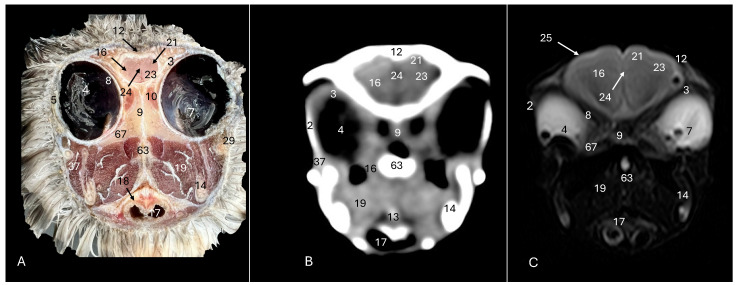
Anatomical cross-section (**A**), soft tissue CT window (**B**) and T2-weighted MR (**C**) transverse images of the Cory’s shearwater head at the level of the *hyperpallium,* corresponding to line II in Figure 1. 2: Sclerotic ossification; 3: sclera; 4: *camera vitrea bulbi*; 5: cornea; 7: *pecten oculi*; 8: retina; 9: *septum interorbitalis*; 10: extraocular muscles; 12: *os frontale*; 13: pharynx; 14: mandible; 16: cerebral hemisphere; 17: trachea; 18: tracheal ring; 19: *musculus pterygoideus pars ventralis*; 21: *Hyperpallium*; 23: *vallecula telencephali*; 24: *fissura longitudinalis cerebri*; 25: meninges; 29: *processus postorbitalis*; 37: *musculus adductor mandibulae externus*; 63: parasphenoid rostrum; 67: *os pterygoideus*.

**Figure 4:**
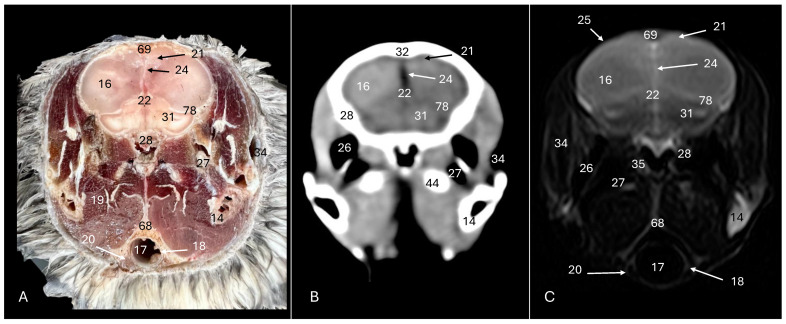
Anatomical cross-section (**A**), soft tissue CT window (**B**) and T2-weighted MR (**C**) transverse images of the Cory’s shearwater head at the level of the optical lobes, corresponding to line III in Figure 1. 14: Mandible; 16: cerebral hemisphere; 17: trachea; 18: tracheal ring; 19: *musculus pterygoideus pars ventralis.* 20: *musculus tracheolateralis*; 21: *hyperpallium*; 22: 3rd ventricle; 24: *fissura longitudinalis cerebri*; 25: meninges; 26: middle ear; 27: tympanic bulla; 28: basioccipital bone; 31: optical lobe; 32: *os parietale*; 34: external acoustic canal; 35: inner ear; 44: otic bone; 68: cricoid cartilage; 69: interparietal bone; 78: tentorial process.

**Figure 5:**
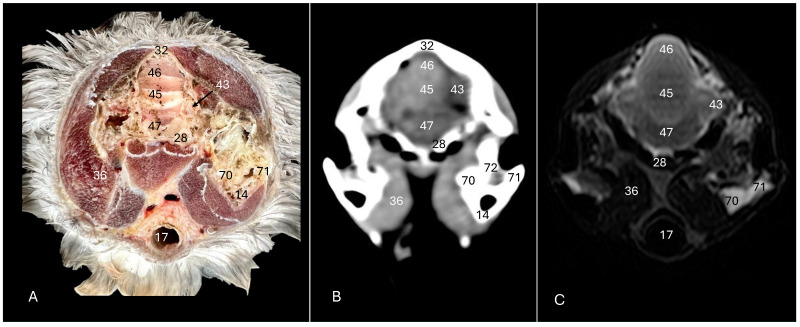
Anatomical cross-section (**A**), soft tissue CT window (**B**) and T2-weighted MR (**C**) transverse images of the Cory’s shearwater head at the level of the *cerebellum,* corresponding to line IV in Figure 1. 14: Mandible; 17: trachea; 28: basioccipital bone; 32: *os parietale*; 36: *musculus depressor mandibulae pars superficialis* plus *musculus adductor mandibulae externus*; 43: cerebellar hemisphere; 45: *cerebellum* (body); 46: folia of *cerebellum*; 47: *pons*; 70: medial process of the mandible; 71: lateral process of the mandible; 72: quadrate articular fossa.

**Figure 6:**
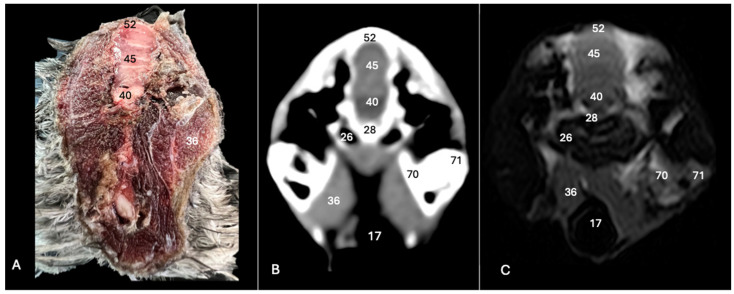
Anatomical cross-section (**A**), soft tissue CT window (**B**) and T2-weighted MR (**C**) transverse images of the Cory’s shearwater head at the level of the caudal *cerebellum,* corresponding to line V in Figure 1. 17: Trachea; 26: middle ear; 28: basioccipital bone; 36: *depressor mandibulae pars superficialis* plus *musculus adductor mandibulae externus*; 45: *cerebellum* (body); 40: *medulla oblongata*; 52: *os supraoccipitale*; 70: medial process of the mandible; 71: lateral process of the mandible.

**Figure 7:**
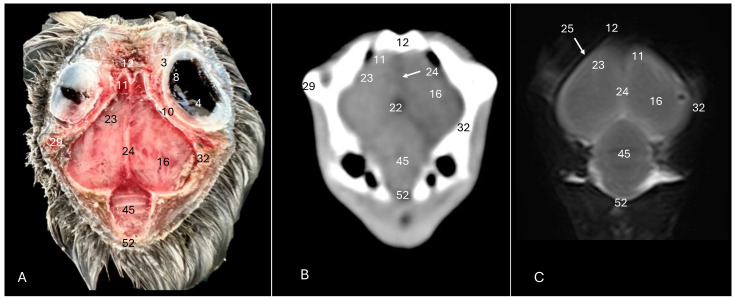
Anatomical cross-section (**A**), soft tissue CT window (**B**) and T2-weighted MR (**C**) dorsal images of the Cory’s shearwater head at the level of the dorsal cerebral hemispheres, corresponding to line VI in Figure 1. 3: Sclera; 4: *camera vitrea bulbi*; 10: extraocular muscles; 11: olfactory bulb; 12: *os frontale*; 16: cerebral hemisphere; 22: 3rd ventricle; 23: *vallecula telencephali*; 24: *fissura longitudinalis cerebri*; 25: meninges; 29: *processus postorbitalis*; 32: *os parietale*; 45: *cerebellum* (body); 52: *os supraoccipitale*.

**Figure 8:**
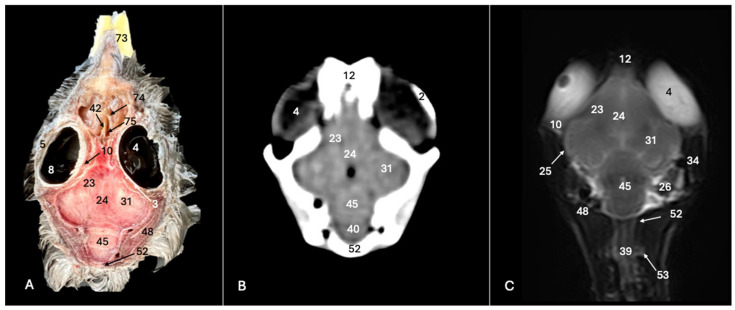
Anatomical cross-section (**A**), soft tissue CT window (**B**) and T2-weighted MR (**C**) dorsal images of the Cory’s shearwater head at the level of the ventral cerebral hemisphere, corresponding to line VII in Figure 1. 2: Sclerotic ossification; 3: sclera; 4: *camera vitrea bulbi*; 5: cornea; 8: retina; 10: extraocular muscles; 12: *os frontale*; 23: *vallecula telencephali*; 24: *fissura longitudinalis cerebri*; 25: meninges; 26: middle ear; 31: optical lobe; 34: external acoustic canal; 39: *medulla spinalis*; 40: *medulla oblongata*; 42: nasal septum; 45: *cerebellum* (body); 48: *musculus constrictor colli*; 52: *os supraoccipitali*; 53: cervical vertebra; 73: beak; 74: *concha nasalis media*; 75: *concha nasalis caudalis*.

**Figure 9:**
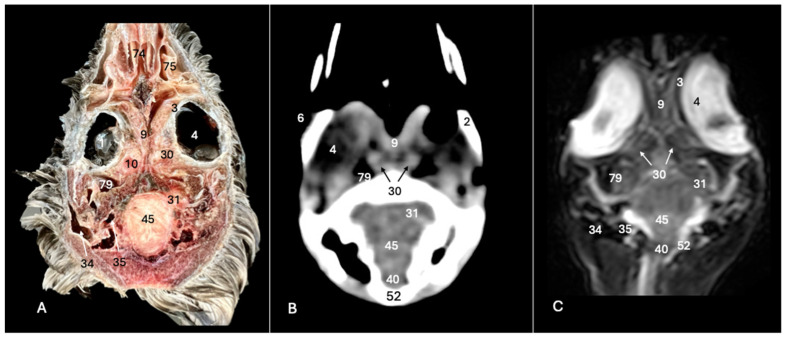
Anatomical cross-section (**A**), soft tissue CT window (**B**) and T2-weighted MR (**C**) dorsal images of the Cory’s shearwater head at the level of the optic nerve, corresponding to line VIII in Figure 1. 1: *Lent cristali*; 2: sclerotic ossification; 3: sclera; 4: *camera vitrea bulbi*; 6: *camera anterior bulbi*; 9: *septum interorbitalis*; *10: extraocular muscle*; 30: optic nerve; 31: optical lobe; 34: external acoustic canal; 35: inner ear; 40: *medulla oblongata*; 45: *cerebellum* (body); 52: *os supraoccipitali*; 74: *concha nasalis media*; 75: *concha nasalis caudalis*; *79:* pneumatization of the temporal bone.

**Figure 10:**
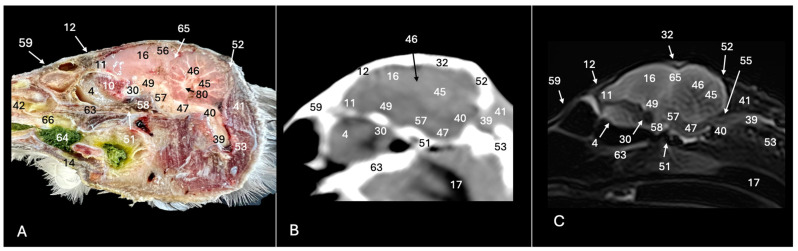
Anatomical cross-section (**A**), soft tissue CT window (**B**) and T2-weighted MR (**C**) sagittal images of the Cory’s shearwater head at the level of the *fissura longitudinalis cerebri*. 4: *Camera vitrea bulbi*; 10: extraocular muscles; 11: olfactory bulb; 12: *os frontale*; 14: mandible; 16: cerebral hemisphere; 17: trachea; 30: optic nerve; 32: *os parietale*; 39: *medulla spinalis*; 40: *medulla oblongata*; 41: *musculus rectus* (*musculus rectus dorsalis* plus *musculus rectus ventralis* plus *musculus rectus lateralis*). 42: nasal septum; 45: *cerebellum* (body); 46: folia of *cerebellum*; 47: *pons*; 49: *diencephalon*; 51: *basis cranii*; 52: *os supraoccipitale*; 53: cervical vertebra; 55: fourth ventricle; 56: *area hipocampalis*; 57: *mesencephalon*; 58: hypophysis; 59: *os nasale*; 63: parasphenoid rostrum; 64: ingesta; 65: *glandula pinealis*; 66: maxilla; 80: internal white substance.

This investigation delved into the intricate anatomy of the Cory’s shearwater CNS and associated structures. We present ten figures that mainly correspond to anatomical cross-sections and CT and T2W MR images (Figure 1, Figure 2, Figure 3, Figure 4, Figure 5, Figure 6, Figure 7, Figure 8, Figure 9 and Figure 10). T2-weighted magnetic resonance imaging images were selected based on their relevance to identifying principal anatomical structures of the brain. The similarity of the sections was prioritized to achieve a better resemblance between the computed tomography and magnetic resonance imaging studies. In addition, we complemented the images with anatomical cross-sections for enhanced clarity and understanding. A pivotal representation of this synergy is depicted in Figure 1, where a sagittal view illustrates the transverse (A) and dorsal (B) orientations, aligning with the anatomical sections delineated in Figure 2, Figure 3, Figure 4, Figure 5, Figure 6, Figure 7, Figure 8, Figure 9 and Figure 10. In this context, the transverse and dorsal section levels (I–VIII) were meticulously aligned, promoting a seamless correlation between the imaging data and the anatomical contexts. Finally, Figure 10 depicts a mid-sagittal anatomical section and the corresponding CT and T2W images, displaying the main structures that compose the head of this bird, especially those related to the CNS and its associated structures.

### 3.1. Anatomical Sections

The anatomical sections utilized in this study proved invaluable for distinguishing the various structures comprising the central nervous system and associated structures. Consequently, we were able to discern the *cerebrum* (*telencephalon*) and its telencephalic hemispheres, located under the frontal bone (labelled with number 16 in Figure 3A, Figure 4A, Figure 7A and Figure 10A), delineated by the longitudinal cerebral fissure (Figure 2A, Figure 3A, Figure 4A, Figure 7A and Figure 8A). Furthermore, transverse cross-sections provided valuable insights into additional features. These included the identification of a slightly caudolaterally positioned rostral groove, known as the *vallecula telencephalic*, as well as the *hyperpallium,* which were prominently displayed on the dorsal surface of each hemisphere (labelled as numbers 23 and 21, respectively, in Figure 3A and Figure 7A). Additionally, these sections revealed the presence of small, pointed olfactory bulbs at the rostral pole of each hemisphere (identified as number 11 in Figure 2A, Figure 7A and Figure 10A). The diencephalon was unmistakably recognized as a continuation rostrally from the *mesencephalon*, which represents the rostral limit of the brain stem and rests on the *basis cranii* (represented as numbers 57 and 51 in Figure 10A). The dorsal and sagittal anatomical images facilitated the identification of the course of the optic nerve penetrating the sclera (labelled as number 30 in Figure 9A and Figure 10A). In addition, the dorsal sections display excellent detail of the optic chiasm located rostrally to the *hypophysis,* which rested on the *basis cranii* (identified as number 58 in Figure 10A). The sagittal section allowed us to identify the pineal gland, an important structure located near the dorsal surface of the diencephalon. It is typically small and appears as a flattened, oval-shaped structure identified ventrally to the parietal bone (labelled as number 65 in Figure 10A). It is a part of the avian endocrine system that plays a role in regulating various physiological processes, including the circadian rhythm and reproductive functions.

These sections also provided insights into other components of the Cory’s shearwater brain. Among these were the *mesencephalon*, the notable large *corpus cerebelli*, the internal medullary body characterized by an internal white substance, and caudally, the small paired cerebellar hemispheres, which were identified in the transverse and dorsal sections (illustrated as numbers 45, 80 and 43, respectively, in Figure 5A, Figure 6A, Figure 7A, Figure 8A, Figure 9A and Figure 10A). Additionally, these sections facilitated the identification of the ventral portion of the *rhombencephalon*, encompassing different components such as the *pons* and the *medulla oblongata*, which rested on the basioccipital bone (illustrated as numbers 47 and 40, respectively, in Figure 5A, Figure 6A and Figure 10A).

Moreover, our examination facilitated a meticulous eye delineation, including various ocular components and enabling a deeper understanding of avian ocular anatomy. Noteworthy elements such as the cornea, sclera, retina, vitreous chamber and lens were precisely identified (displayed as numbers 5, 3, 8, 4, 6 and 1, respectively in Figure 2A, Figure 3A, Figure 7A, Figure 8A, Figure 9A and Figure 10A). Simultaneously, our investigation uncovered many associated structures surrounding the avian eyeball, unveiling the intricate network of anatomical support and functionality. Furthermore, associated eyeball structures, including the interorbital septum and extraocular muscles, were among the structures distinguished, which help in maintaining the structural integrity and functionality of the avian eye (illustrated as numbers 9 and 10 as depicted in Figure 2A, Figure 3A, Figure 7A, Figure 8A, Figure 9A and Figure 10A). 

Regarding the morphology of the skull and its diverse bony structures, these sections offered crucial insights. Thus, the examination revealed detailed information about various components, including the nasal, frontal, parietal, temporal, otic, occipital (basioccipital and supraoccipital), pterygoid, interparietal and mandible/maxillary bones (illustrated as numbers 59, 12, 32, 79, 44, 28, 52, 67, 69, 14 and 66, respectively in Figure 2A, Figure 3A, Figure 4A, Figure 5A, Figure 6A, Figure 7A, Figure 8A, Figure 9A and Figure 10A). Additionally, the utilization of these sections proved indispensable in discerning the structure of both the roof of the oral cavity and the pharynx and the trachea (represented as numbers 13 and 17 in Figure 2A, Figure 3A, Figure 4A, Figure 5A and Figure 10A). 

Furthermore, various head muscles, including the musculus tterygoideus pars ventralis, musculus tracheolateralis, musculus rectus capitus (comprising the musculus rectus dorsalis, musculus rectus ventralis and musculus rectus lateralis), musculus depressor mandibulae pars superficialis, musculus adductor mandibulae externus and constrictor colli were distinguished (labelled as numbers 19, 20, 41, 36, 37 and 48, respectively in Figure 2A, Figure 3A, Figure 4A, Figure 5A, Figure 6A, Figure 8A and Figure 10A).

### 3.2. Computed Tomography (CT)

In terms of the skull, the transverse, dorsal and sagittal CT scans provided clear delineation of the bones that encompasses it as the nasal, frontal, parietal, otic, occipital and mandible/maxilla bones, displaying high CT density with the soft tissue window settings (labelled as numbers 59, 12, 32, 44, 28, 52, 14 and 66, respectively, in Figure 2B, Figure 3B, Figure 4B, Figure 5B, Figure 6B, Figure 7B, Figure 8B, Figure 9B and Figure 10B). In addition, these CT images displayed various components of the encephalon, which presented a moderate or intermediate CT density and appeared grey. Therefore, the transverse CT image showed two telencephalic hemispheres, separated by the *fissura longitudinalis cerebri* (identified as number 24 in Figure 2B, Figure 3B, Figure 4B, Figure 7B, Figure 8B and Figure 10B). These included the identification of the *vallecula telencephali*, prominently visible on the dorsal surface of each hemisphere (cited as number 23 in Figure 3B, Figure 7B and Figure 8B). Additionally, these sections depicted the presence of the pointed olfactory bulbs, which showed moderate attenuation (shown as number 11 in Figure 2B, Figure 7B and Figure 10B). Furthermore, other structures of the CNS displaying intermediate attenuation were identified, including the *hyperpallium*, the *cerebellum*, the optic lobe and the brain stem (depicted as numbers 21, 45, 31, 47 and 40, respectively, in Figure 3B, Figure 4B, Figure 5B, Figure 6B, Figure 7B, Figure 8B and Figure 10B).

Additionally, we identified diverse components of Cory´s shearwater eyeballs. Therefore, we distinguished the dorsal and ventral arch of the sclerotic ossifications and the lens, appearing as hyperattenuating structures just posterior to the iris (labelled as numbers 2 and 1, respectively, in Figure 2B, Figure 3B, Figure 8B and Figure 9B). In contrast, other elements, such as the sclera or the vitreous chamber, were accurately pinpointed due to their moderate or low CT attenuation (represented as numbers 3 and 4, respectively, in Figure 2B, Figure 3B, Figure 8B and Figure 9B).

### 3.3. Magnetic Resonance Imaging (MRI)

No discernible anatomical differences were observed in the examined shearwaters; however, it is noteworthy that compared to CT, CNS structures were better visualized. Therefore, T2W MR images demonstrated precise alignment with the cranial structures observed in the cadaveric cross-sections, providing a comprehensive view of the CNS and its associated structures. Thus, the examination of Cory’s shearwater brains in transverse, dorsal and sagittal planes allowed the observation of the two telencephalic hemispheres, revealing regions of a moderate-intensity signal (labelled as number 16 in Figure 2C, Figure 3C, Figure 4C, Figure 7C, Figure 8C and Figure 10C). Notably, these imaging planes also facilitated the precise identification of the olfactory bulb, characterized by its small, tapering rostral structure, with moderate and uniform signal intensity (depicted as number 11 in Figure 2C, Figure 7C and Figure 10C). Moreover, the transverse T2W MR images displayed two curved dorsal structures with moderate signal intensity, corresponding with the *hyperpallium* (corresponding to number 21 in Figure 3C and Figure 4C). The *diencephalon*, another constituent of the forebrain, exhibited limited differentiation from the adjacent *mesencephalon*, displaying signals of moderate to low intensity (identified as numbers 49 and 57, respectively, in Figure 10C). Moreover, notable features of the mesencephalon, such as the optic lobe, were perfectly discernible in the transverse and dorsal T2-weighted MRI images, showcasing similar signal characteristics (represented as number 31 in Figure 4C and Figure 8C). Furthermore, other crucial components of the central nervous system, such as the *pons* and the *medulla oblongata,* were identifiable across transverse, dorsal and sagittal planes, and characterized by low-intensity signals (depicted as numbers 47 and 40, respectively, in Figure 5C, Figure 6C and Figure 10C). Adjacent to the brain stem, the *corpus cerebellum* and the small paired cerebellar hemispheres displayed indistinct areas of hypo- and moderate-intensity signals (shown as numbers 45 and 43, respectively, in Figure 5C, Figure 6C, Figure 7C, Figure 8C, Figure 9C and Figure 10C).

Concerning CNS-associated structures, a variety of the *bulbus oculi* structures were depicted. Subsequently, the vitreous chamber consistently exhibited hyperintense signals across all planes examined (labelled as number 4 in Figure 2C, Figure 3C, Figure 8C, Figure 9C and Figure 10C). However, the cornea, sclera, lens and sclerotic ossifications displayed a hypointense signal in these MR images (corresponding to numbers 5, 3, 1 and 2, respectively, in Figure 2C, Figure 3C and Figure 8C). Furthermore, various head muscles, such as the *musculus pterygoideus pars ventralis, musculus tracheolateralis, musculus rectus capitus (musculus rectus dorsalis* plus *musculus rectus ventralis* plus *musculus rectus lateralis*), *musculus adductor mandibulae externus* and *constrictor colli* exhibited signals of intermediate intensity in the T2-weighted MR images (represented as numbers 19, 20, 41, 37 and 48, respectively, in Figure 2C, Figure 3C, Figure 4C, Figure 8C and Figure 10C).

## 4. Discussion

To the authors’ knowledge, this is the first description of a Cory’s shearwater head using anatomical cross-sections and their corresponding CT and MR images. In avian medicine, conventional radiography has historically been a fundamental tool for diagnostic imaging, providing valuable information on musculoskeletal and respiratory processes and alterations in the coelomic cavity [31,36]. However, its utility is limited in the cranial region due to structure overlap and low resolution [30,31]. Despite modern imaging modalities presenting more promising options capable of offering precise anatomical and pathological information, their use remains challenging due to the high economic cost of these types of equipment, the need for animal sedation and possibly restraining the animal, and the duration of the examination in the case of MR studies [32,33,34,35,36,37,38]. Other imaging equipment, such as the micro-CT, offers images with higher spatial resolution and thinner slice thickness than those obtained with conventional CT scanners. Nonetheless, this equipment is not usually available in veterinary hospitals [45].

Throughout this study, we selected CT and MR images that better matched the anatomical cross-sections. The acquisition of adequate-resolution images of the central nervous system in this study was enhanced by the physical volumetric factor of the Cory’s shearwater brain. In other words, animals with larger brain volumes tend to exhibit better resolution in the obtained images. This relationship between brain size and the quality of acquired images is particularly relevant when considering the strength of the equipment. In our study, we utilized a 1.5 Tesla magnet, which has certain limitations compared to previous research employing higher field magnets, specifically 3 and 4.7 Tesla magnets [30,31,32,33,34,35,36]. Our investigation has demonstrated a significant improvement in image resolution, especially when compared with other studies on seabirds using similar strength fields, such as Atlantic puffins, that present small head dimensions, leading to the more limited resolution of MR images [46]. Despite this, our results displayed images with similar resolutions to other studies on larger birds such as the red kite, common buzzard, or African grey parrot [30,31,32,33,34,35,36]. The significance of this finding lies in a deeper understanding of the factors influencing the quality of central nervous system images in animals. By acknowledging the relationship between brain sizes, the imaging technique utilized and the resolution obtained, we can optimize future studies to ensure greater precision in evaluating brain structures across different species.

As reported in recent studies [30,32,46], modern imaging diagnostic techniques showed adequate detail of the different regions of the avian brain. Here, both techniques allowed the observation of specific formations within the telencephalic hemispheres, such as the *vallecula telencephali*, the sagittal eminence observed as a prominence lateral to the *fissura longitudinalis cerebri*, the olfactory bulb and a prominent *hyperpallium.* This last formation is the homologue of the mammalian neocortex involved in visual capabilities and spatial cognition [47,48,49]. Further studies of the *hyperpallium* across multiple bird species, mainly birds of prey, should be performed to compare their development. 

Concerning the Cory’s shearwater mesencephalon, it was easily identified through CT and T2W MR images. Thus, the basioccipital bone was used as a landmark to visualize the optic lobes in the CT images, whereas their caudoventral situation related to the telencephalon and the identification of the tentorial process were pivotal to observing them with T2W images. These lobes were quite prominent mainly due to their visual, auditory and somatosensory functions [30,46,49]. As reported in other studies [30,50], the largest optic lobes have been identified in birds of prey. However, further investigations comparing these birds with seabirds are necessary since important developments of these structures, and optic nerves, as well as the optic chiasm, have also been observed on them [43,46].

Sagittal T2W images were essential to observing the large volume of the cerebellum, and its situation above the brain stem, which did not present a clear distinction between *pons* and *medulla oblongata,* as happens in mammals [30,43]. The combination of anatomical cross-sections and transverse T2W images displayed the cerebellar hemispheres. This finding contrasts with other studies using higher-strength field magnets, where these structures were not easily depicted [30]. 

As reported in other investigations performed on birds, felids and reptiles, the dorsal, transverse and sagittal anatomical sections offered intricate insights into the different elements and shapes of the avian eyeball [51,52,53]. It was characterized by its significant size relative to cranial volume and its lateral positioning, inherent to avian anatomy, showing a distinctive globose morphology with a subtle medial flattening. In addition, the incorporation of CT and T2W images confirmed the visualization of the vitreous chamber, the sclera, the extraocular muscles, the lens and the sclerotic ossifications. These ossifications were distinguishable in the CT images that depicted the dorsal and ventral arches. Reports of this structure have stated its association with the visual capabilities and activity patterns of seabird species [46].

In contrast to other studies performed on birds, the combination of CT and MR images was essential in the visualization of the shape and the different bones that comprise the bird skull. Employing a soft tissue CT window allowed us to distinguish its dome shape, and how it was robust and flattened compared to the skull of other seabird species [46]. These bones showed relevant pneumatization that could be related to their living and foraging ecology, mainly focused on small fishes and squids, which were captured by shallow immersions [2,3,4]. In addition, this CT window was quite helpful in displaying with adequate resolution the eyes and the nervous structures from the remaining tissues. 

This approach has proven invaluable in evaluating the anatomical intricacies of the CNS and its related structures across a diverse range of wildlife species. From reptiles to rodents, terrestrial mammals to exotic avian species like the red kite, common buzzard and common kestrel, as well as domestic birds such as the pigeon and the African grey parrot, MRI has played a crucial role in identifying CNS components [46,52,54,55,56,57]. Unlike traditional imaging methods, modern imaging diagnostic techniques allow detailed images from various anatomical perspectives without specimen repositioning [46,51,52,54,55,58]. The unparalleled ability of MRI to discern between cranial bones and soft tissues makes it a superior tool for assessing the CNS and associated structures compared to computed tomography. However, we should highlight the limitations related to the use of carcasses, which were mainly observed in the MR images due to changes in the signal intensity of some tissues. Hence, it would be interesting to perform future investigations using adult and live specimens to assess these changes and the differences in the resolution and volume of the CNS and associated structures.

## 5. Conclusions

The application of these modern imaging techniques combined with anatomical cross-sections not only reveals the detailed anatomy of Cory’s shearwater but also provides a platform for a better understanding of the CNS and its related structures, contributing to advancing knowledge in the fields of ornithology and comparative neuroscience.

## Data Availability

The data supporting reported results can be found at “accedacris.ulpgc.es”.
